# Association between adult body shape index and serum levels of the anti-aging protein Klotho in adults: a population-based cross-sectional study of the NHANES from 2007 to 2016

**DOI:** 10.3389/fendo.2025.1424350

**Published:** 2025-02-11

**Authors:** Li Gong, Jinghan Xu, Yiyang Zhuang, Liwei Zeng, Zhenfei Peng, Yuzhou Liu, Yinluan Huang, Yutian Chen, Fengyi Huang, Chunli Piao

**Affiliations:** ^1^ Department of Diabetes, Shenzhen Bao’an District Hospital of Traditional Chinese Medicine, Guangzhou University of Chinese Medicine, Shenzhen, China; ^2^ Department of Endocrinology, Shenzhen Hospital (Futian) of Guangzhou University of Chinese Medicine, Shenzhen, China; ^3^ Department of Geriatrics, Shenzhen Bao’an District Hospital of Traditional Chinese Medicine, Guangzhou University of Chinese Medicine, Shenzhen, China

**Keywords:** adult body shape index, Klotho protein, national health and nutrition examination survey, obesity, cross-sectional study

## Abstract

**Purpose:**

Adult body shape index (ABSI) is widely recognized as a reliable indicator for evaluating body fat distribution and dysfunction. However, the relationship between ABSI and Klotho protein, known for its anti-aging biological function, has not yet been investigated. Therefore, the aim of this study was to assess the correlation between ABSI and serum Klotho levels in adults residing in the United States.

**Methods:**

A cross-sectional study of participants was conducted based on the 2007–2016 National Health and Nutrition Examination Survey. Visceral adiposity was determined using the ABSI score, and Klotho protein concentration was measured using an enzyme-linked immunosorbent assay kit. Multiple regression models were used to estimate the association between ABSI and Klotho protein after adjusting for several potential confounding variables. Subgroup analysis of ABSI and Klotho was performed using restricted cubic splines.

**Result:**

A total of 11,070 adults were eligible for participation, with a mean ABSI of 8.28 ± 0.45 and a mean Klotho protein concentration of 853.33 ± 309.80 pg/mL. Multivariate regression analysis showed that participants with high ABSI scores had lower serum Klotho protein concentrations. When ABSI was divided into quartiles, after full adjustment, Klotho protein levels were lower in participants in the fourth fully adjusted ABSI quartile (Q4: -0.352 pg/ml) than in those in the lowest quartile (Q1) (P<0.0001).

**Conclusion:**

There was a negative linear correlation between ABSI score and serum Klotho concentration. Higher ABSI was associated with lower serum Klotho concentrations; however, this association did not seem to be significant in subjects with BMI ≥30 kg/m2.Further study is needed to verify the causality of this association and elucidate the underlying mechanisms.

## 1 Introduction

In recent years, obesity has emerged as an independent risk factor for various diseases, significantly impacting life expectancy worldwide (1). Body mass index (BMI) and waist circumference (WC) are the commonly used indicators of body size; however, they have certain limitations. Adult body shape index (ABSI) is a novel anthropometric index that considers abdominal circumference, height, and weight to better reflect individual fat distribution and visceral fat proportion, compared with BMI (2). ABSI proves to be a superior predictor of cardiovascular disease risk when compared with WC (3–6), as well as mortality associated with central obesity. Studies on nutrition have demonstrated that ABSI and its changes can independently predict all-cause mortality in the elderly Chinese population (7). Moreover, a strong positive correlation exists between ABSI and directly measured visceral fat content (8, 9), making it a potential surrogate indicator for arterial stiffness in patients with type 2 diabetes mellitus. By replacing WC with ABSI in the diagnostic criteria for metabolic syndrome, one can more effectively identify individuals at risk of renal function decline and arterial stiffness (10). Furthermore, ABSI provides a more accurate description of changes in circulating insulin and lipoproteins than traditional obesity indicators (11). Studies have confirmed consistent exposure-response relationships between ABSI and all-cause/cardiovascular mortality in cohorts of Asian patients with diabetes (12).

In 1997, Kuro-o et al. (13) discovered that mice with deficient Klotho gene expression exhibited premature aging syndromes and a shortened lifespan, confirming the role of Klotho as an aging regulator. The Klotho gene produces α-Klotho, β-Klotho, and γ-Klotho proteins; however, “Klotho” typically refers to α-Klotho (14). Reportedly, α-Klotho binds to the ligand domain of the fibroblast growth factor receptor and then binds to FGF-23 to exert biological effects (15). Both α-Klotho and β-Klotho are crucial components of the endocrine fibroblast growth factor receptor complex. Targeting the FGF-Klotho endocrine axis plays a critical role in the pathophysiology of aging-related diseases, such as diabetes, cancer, arteriosclerosis, and chronic kidney disease (16), some of which are associated with obesity (e.g., arteriosclerosis, diabetes, osteoporosis) (17, 18). Additionally, Klotho protein expression improves vascular calcification by increasing autophagy, which is beneficial for vascular-related diseases (19–21). Furthermore, Klotho appears to be involved in regulating phosphate homeostasis and insulin signaling while also inhibiting oxidative stress, thereby participating in glucose and lipid metabolism (22). Additionally, there is a correlation between serum Klotho concentration and age in humans (23). A study revealed that cerebrospinal fluid levels of Klotho were significantly negatively correlated with body weight/BMI due to the central involvement in obesity’s pathological process (24), while soluble Klotho concentration was inversely correlated with abdominal obesity/high triglycerides (14). Recently, a nonlinear relationship between visceral adiposity index (VAI) score and serum concentration of the anti-aging protein Klotho has been reported. This suggests a potential direct involvement between Klotho expression level and obesity/aging relationships (25).

The ABSI index is a new measure used to assess an individual’s body size and health risk, which combines data from three dimensions: weight, height, and waist circumference. Previous studies (7, 10) have mainly explored the association between ABSI and metabolic and cardiovascular diseases; However, its relationship with Klotho protein has not been thoroughly examined. Considering that obesity may lead to a reduced metabolic rate and is associated with an increased risk of multiple chronic diseases (12), thereby promoting aging and shortened life expectancy, it seems particularly important to investigate the association between ABSI index and Klotho protein. Therefore, in-depth study of the relationship between ABSI index and Klotho protein will not only help us to more fully understand the possible pathogenesis of aging, but also provide new perspectives and strategies for the prevention of aging. Therefore, we used the National Health and Nutrition Examination Survey (NHANES) data to analyze the association between ABSI and Klotho protein levels, aiming to provide new ideas about its mechanism. We hypothesized that higher ABSI would be associated with lower serum Klotho protein concentrations.

## 2 Materials and methods

### 2.1 Study design and population

The information in this study was based on the NHANES data collected from 2007 to 2016. NHANES is a research program designed to investigate the health and nutritional status of participants in the United States, comprising interviews, examinations, and laboratory components. The data from 2007 to 2016 included five consecutive cycles, totaling 13,766 cases. There were 96,862 ABSI cases initially. Subsequently, 85,792 participants without information on ABSI or Klotho protein data were excluded from this study. After performing sensitivity analysis, 11,070 eligible participants were included for further analysis. Written informed consent was obtained for all study protocols included in this study, and the research was approved by the research ethics review board of the National Center for Health Statistics.

### 2.2 Outcome and exposure factors

The main exposure factor was ABSI, calculated using the following formula:


$$\text{ABSI}=\frac{WC(\text{cm})}{\sqrt{Height(cm)}•\sqrt[{3}]{{\text{BMI}}^{2}}}$$

Where WC was expressed in cm and BMI in kg/m^2^.

The primary outcome was serum Klotho concentration. Serum samples were collected from participants, transferred, and stored at –80°C. Klotho concentrations were determined using a commercially available enzyme-linked immunosorbent assay kit produced by IBL International, Japan. The sensitivity level of the assay was 6 pg/mL. All study samples were run in duplicate, bisected, and measured separately. The mean of the two concentration values was calculated as the result.

### 2.3 Covariates

Data regarding additional covariates were collected from each cycle of the NHANES. Continuous variables included age and poverty-to-income ratio (PIR). Categorical variables included sex, age, race, education level, marital status, smoking, alcohol consumption, hypertension, and diabetes. Based on previous studies, we adjusted for several possible confounding variables. The variables were divided into different categories: age: 40–59 years and ≥60 years; BMI:<25, 25-30, and ≥30 kg/m^2^; PIR: ≤1.3, >1.3 and ≤3.5, >3.5%, or missing; race: Mexican American, other Hispanic, non-Hispanic White, non-Hispanic Black, and other races; marital status: married and other; education level: below high school, grades 9–12, and above high school; smoking status, alcohol consumption status, and self-reported medical conditions, including diabetes and hypertension: yes or no. When the missing covariate value exceeded 2% of the total population, dummy variables were used instead.

### 2.4 Statistical analysis

**Table 1** clearly describes the baseline characteristics of all participants by proportions or means ± standard errors (SE). Categorical variables were analyzed using weighted chi-square analysis, and continuous variables were evaluated using a weighted linear regression model. ABSI was treated not only as a continuous independent variable but also as a categorical variable, divided into quartiles, with the lowest quartile as the reference. To investigate the independent relationship between ABSI and Klotho protein, we performed a multivariate generalized linear regression analysis. Model 1 was not adjusted. Model 2 was adjusted for age, sex, and race. Model 3 was adjusted for sex, age, race, PIR, BMI, education, marital status, smoking, alcohol consumption, diabetes, and hypertension to allow for further subgroup analysis and explore potential nonlinear associations. All statistical analyses were conducted using R version 3.6.3 and EmpowerStats. A P-value<0.05 was considered statistically significant (two-tailed).

**Table 1:** Characteristics of participants by categories of adult body shape index in NHANES 2007–2016^ab^.

Characteristic	All	ABSI quartiles				P-value
		Q1(6.10-7.99)	Q2(7.99-8.28)	Q3(8.28-8.57)	Q4(8.57-11.10)	
No. of participants	11070	2812	2713	2752	2793	
Adult body shape index	8.28 ± 0.45	7.711 ± 0.243	8.141 ± 0.082	8.421 ± 0.084	8.842 ± 0.236	<0.0001
Klotho concentration (pg/ml)	853.33 ± 309.80	893.589 ± 331.977	852.179 ± 313.726	844.986 ± 299.446	822.153 ± 287.954	<0.0001
Age (years)	57.62 ± 10.83	52.862 ± 9.816	55.664 ± 10.238	58.598 ± 10.228	63.343 ± 10.043	<0.0001
40-59 (%)	54.45	18.509	15.393	12.520	8.121	
≥60 (%)	45.55	6.893	9.115	12.340	17.109	
Sex (%)						<0.0001
Female	50.94	16.893	12.285	10.894	10.867	
Male	49.06	8.509	12.222	13.966	14.363	
Race (%)						<0.0001
Mexican American	15.31	3.279	4.146	4.038	3.749	
Other Hispanic	10.87	2.692	2.918	2.773	2.484	
Non-Hispanic white	45.40	9.874	10.136	11.454	13.939	
Non-Hispanic black	19.62	7.218	4.932	4.246	3.225	
Other races	8.80	2.240	2.376	2.349	1.834	
Poverty income ratio	2.65 ± 1.65	2.85 ± 1.67	2.73 ± 1.66	2.63 ± 1.66	2.39 ± 1.57	<0.0001
≤1.3 (%)	29.85	6.531	6.929	7.570	8.817	
>1.3 and ≤3.5 (%)	36.16	8.808	8.835	8.835	9.684	
>3.5 (%)	33.99	10.063	8.744	8.455	6.730	
BMI (kg/m2)						<0.0001
<25 (%)	23.62	6.531	6.929	7.570	8.817	
25-30 (%)	34.94	8.808	8.835	8.835	9.684	
≥30 (%)	41.44	10.063	8.744	8.455	6.730	
Education level (%)						<0.0001
Less than high school	26.41	5.474	5.953	6.902	8.085	
High school	22.32	5.393	5.294	5.574	6.061	
More than high school	51.26	14.535	13.261	12.385	11.084	
Marital Status (%)						<0.0001
Married	60.10	14.824	15.176	15.474	14.625	
Others	39.90	10.578	9.332	9.386	10.605	
Hypertension (%)						<0.0001
Yes	46.01	9.494	10.524	11.689	14.230	
No	53.99	15.908	13.984	13.170	10.930	
Diabetes (%)						<0.0001
Yes	18.11	2.755	3.550	4.878	6.929	
No	81.89	22.647	20.958	19.982	18.302	
Smoking (%)						<0.0001
Yes	49.23	9.874	11.319	12.990	15.050	
No	50.77	15.528	13.189	11.870	10.181	
Alcohol consumption (%)						<0.0001
Yes	71.40	17.407	17.552	18.302	18.139	
No	28.60	7.995	6.947	6.558	7.064	

a Mean ± SE for continuous variables, and a P-value calculated by weighted t-test.

b % for categorical variables, and P-value calculated by weighted Chi-square test.

## 3 Results

### 3.1 Participant characteristics

The baseline characteristics of the participants according to the ABSI category are shown in **Table 1**. A total of 11,070 US adults were eligible to participate in the study. Among them, 50.94% were female and 49.06% were male. The mean ± SE of ABSI was 8.28 ± 0.45. The mean ± SE of Klotho protein concentration was 853.33 ± 309.80 pg/ml. Participants in the fourth ABSI quartile had the lowest serum Klotho protein concentration (Q4: 822.153 ± 287.954pg/ml), compared with those in the other three quartiles (Q1: 893.589 ± 331.977, Q2: 852.179 ± 313.726, and Q3: 844.986 ± 299.446 pg/ml, p≤ 0.0001).

### 3.2 Multivariate regression analysis

**Table 2** shows that, in the unadjusted model [b (95% confidence interval [CI]) = -0.163 (-0.191, -0.136)], the minimum adjustment model [-0.109 (-0.138, -0.080)], and the fully adjusted model [-0.119 (-0.149, -0.089)], ABSI was negatively correlated with Klotho protein concentration. Multivariate regression analysis showed that participants with higher ABSI scores had lower serum Klotho protein concentrations. When ABSI was categorized into quartiles, Klotho protein levels were lower among fully adjusted ABSI participants in the fourth quartile (Q4: -0.352 pg/ml), compared with the levels in participants in the lowest quartile (Q1) (P<0.0001). Significant associations were found between ABSI and Klotho protein levels for each quartile array in all three models (P<0.0001). Klotho protein levels were also significantly lower in models 1, 2, and 3 for participants with higher ABSI scores in the second, third, and fourth quartiles than for those with lower ABSI scores in the first quartile (6.10-7.96), with P for trend<0.0001 for all three models.

**Table 2:** Association between adult body shape index and serum anti-aging protein Klotho.

Exposure	Model 1[^a^]	Model 2[^b^]	Model 3[^c^]
	β (95% CI) P-value	β (95% CI) P-value	β (95% CI) P-value
ABSI	-0.163 (-0.191, -0.136)	-0.109 (-0.138, -0.080)	-0.119 (-0.149, -0.089)
	<0.0001	0.0002	<0.0001
ABSI quartile			
Q1	Ref	Ref	Ref
Q2	-0.387(-0.441, -0.333)	-0.299 (-0.383, -0.215)	-0.313 (-0.398, -0.228)
	<0.0001	0.0004	0.0002
Q3	-0.474 (-0.558, -0.389)	-0.343 ((-0.431, -0.256)	-0.356 ((-0.444, -0.269)
	<0.0001	<0.0001	<0.0001
Q4	-0.481 (-0.566, -0.397)	-0.321 (-0.410, -0.231)	-0.352 (-0.444, -0.260)
	<0.0001	0.0004	0.0001

a Model 1: adjusted for no covariates.

b Model 2: adjusted for age, sex, and race.

c Model 3: adjusted for sex, age, race, poverty income ratio, body mass index, education, marital status, smoking, alcohol use, diabetes, hypertension.

### 3.3 Subgroup analysis

**Figure 1:**
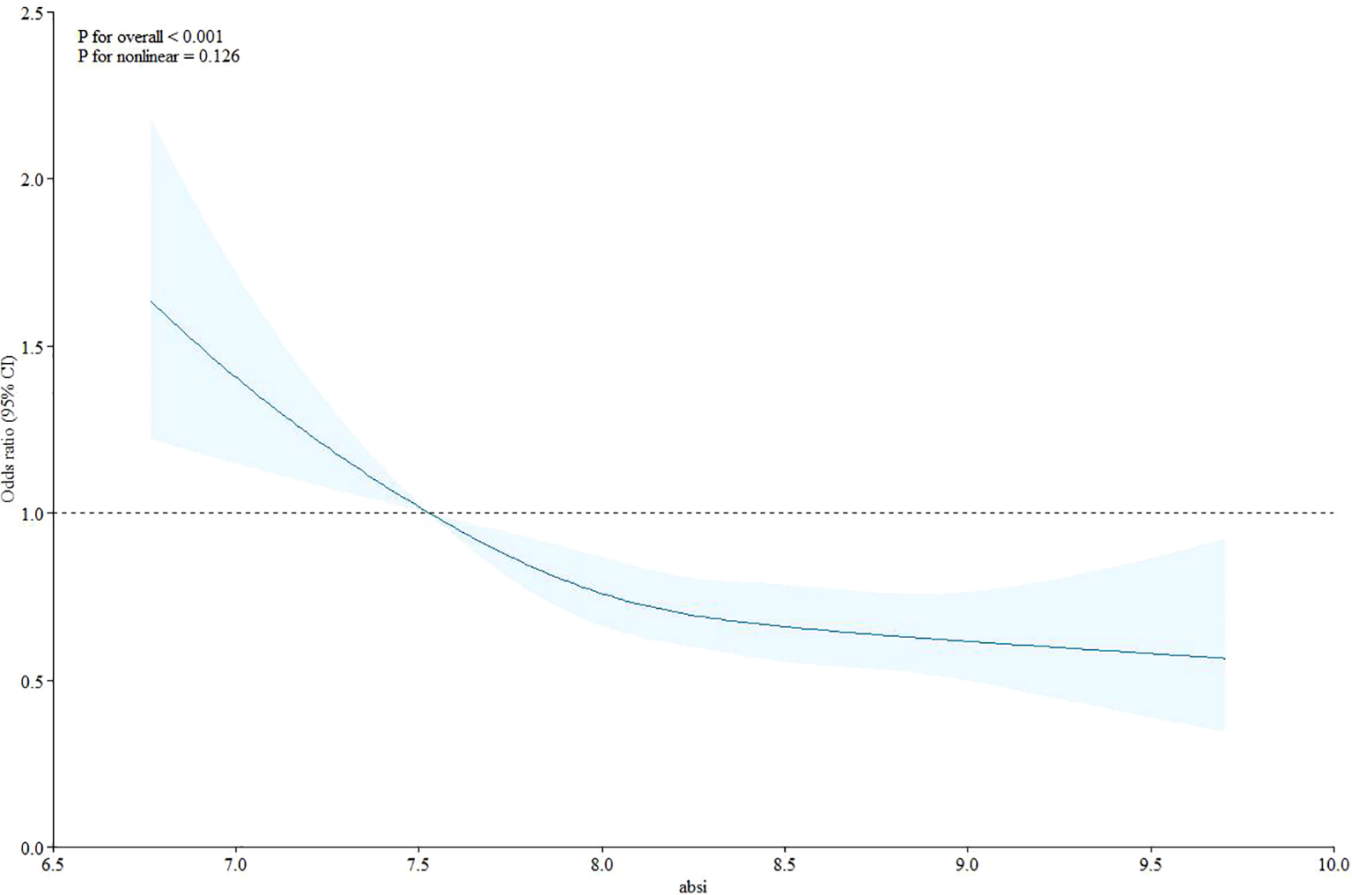
Restricted cubic splines for the association of adult body shape index (ABSI) and serum anti-aging protein.

We performed subgroup analyses, presented as restricted cubic splines, to explore potential nonlinear associations between ABSI and serum Klotho protein concentrations (**Figure 1**, **Table 3**). The fully adjusted restricted cubic spline plot (**Figure 1**) showed no nonlinear relationship between ABSI and Klotho protein levels (P for nonlinearity=0.126). Subgroup analysis showed no significant correlation between Klotho protein level and ABSI when BMI was ≥30 kg/m^2^ (95% CI: 0.85-1.02; P=0.148). Additionally, after adjusting for confounding variables including smoking, gender, age, diabetes, alcohol consumption, and other covariates, a significant inverse association was observed between ABSI and Klotho protein levels.

**Table 3:** Subgroup analysis.

	OR	95%CI	P value
BMI (kg/m^2^)			
<25	0.83	0.74-0.93	<0.001
25-29	0.87	0.78-0.96	0.007
≥30	0.93	0.85-1.02	0.148
Smoking			
No	0.88	0.81-0.95	<0.001
Yes	0.90	0.82-0.99	0.022
Diabetes			
No	0.89	0.84-0.96	<0.001
Yes	0.86	0.76-0.98	0.024
Alcohol consumption			
No	0.91	0.83-1.00	0.048
Yes	0.87	0.81-0.94	<0.001
Sex			
Female	0.88	0.82-0.95	<0.001
Male	0.87	0.79-0.96	0.005
Age (years)			
40-59	0.88	0.82-0.96	0.002
≥60	0.88	0.81-0.96	0.005

Subgroup analyses were adjusted for sex, age, smoking, alcohol use, and diabetes.

## 4 Discussion

To the best of our knowledge, this is the first study to assess the association between ABSI and serum concentrations of the anti-aging protein Klotho by analyzing extensive population data from NHANES. However, there was a significant negative correlation between ABSI and Klotho protein concentration after adjusting for confounding factors, such as smoking, sex, age, diabetes, and alcohol consumption.

In this study, we investigated the correlation between the ABSI index and Klotho protein. In addition, we used subgroup analyses to explore their associations in different populations. We found an inverse correlation between ABSI index and Klotho protein. Under subgroup analysis, we found no significant association between ABSI index and Klotho protein in participants with BMI ≥ 30 kg/m^2^. Previous studies have also explored the association between Klotho protein and several clinicopathological factors (22, 24).The Klotho gene is predominantly expressed in kidney and brain tissues, giving rise to membrane-bound and secreted proteins that function as membrane-bound receptors and humoral regulators, respectively. The biological functions attributed to Klotho can give rise to various physiological effects, as well as diseases, including obesity (18, 19). Several studies have demonstrated an indisputable relationship between increased all-cause mortality and overweight or obesity, as measured by BMI (6). Moreover, ABSI seems to describe changes in circulating insulin and lipoproteins more accurately, compared with BMI (11), and ABSI can help determine the risk of sarcopenia in overweight/obese individuals (26).Previous studies have shown (6) that BMI, as a single quantitative indicator of obesity, cannot distinguish between fat and muscle content and cannot reflect individual fat distribution, while ABSI was selected as an indicator in our study to make up for this deficiency.

Our study found that after adjusting for confounding factors, higher ABSI population was associated with lower Klotho protein concentration. However, this relationship was not evident in individuals with BMI≥30 kg/m 2. This may be based on the difference between BMI and ABSI. Our results show that in the subgroup analysis, ABSI is significantly correlated with Klotho protein in variables such as age, gender, smoking history, drinking history, and diabetes history. Study (26) found a direct and significant association between age and obesity phenotype, with a higher chance of obesity phenotype in women with a history of diabetes and older age; however, it also pointed out that in different models of obesity, current smokers have a lower chance of obesity phenotype than non-smokers. A cross-sectional study (27) proposed that age and smoking are risk factors for cardiovascular and cerebrovascular diseases, while Klotho is a protective factor. Previous studies have shown that there is no significant correlation between serum soluble Klotho and gender, but serum soluble Klotho levels usually decrease with age. These findings prompt us to further raise questions in multiple covariates such as age, gender, smoking history, and diabetes history.

Existing studies have shown that visceral obesity is a risk factor for various metabolic diseases (27). Recent studies have shown that inflammation, both local and systemic, can reduce Klotho expression in the kidneys (28, 29). Therefore, proinflammatory cytokines from adipose tissue may affect serum Klotho protein concentrations. This can explain the possible correlation between ABSI and Klotho protein concentration from the perspective of inflammation.

Recent studies have confirmed that Klotho knockout mice are resistant to obesity induced by a high-fat diet due to a reduction in white adipose tissue, implying an effect of Klotho on adipocyte differentiation and maturation *in vivo* (24). Abnormal expansion of white adipose tissue and abnormal recruitment of adipose precursor cells can not only lead to obesity but also affect glucose metabolism (23). Klotho may affect adipocyte maturation, as well as systemic glucose metabolism. ABSI is not associated with BMI, but it is more likely to reflect central obesity when combined with waist circumference. This is different from previous studies (25) that directly reflect the relationship between visceral fat, and ABSI is more convenient than VAI in terms of measurement methods.

Additionally, previous studies have confirmed a negative correlation between CSF a-Klotho and BMI (24). Similarly, our study revealed a comparable association between serum a-Klotho levels and ABSI. It has been shown that soluble Klotho protein concentration is negatively correlated with the occurrence of metabolic syndrome, as well as abdominal obesity and hypertriglyceridemia (14). Obesity is classified into several types (30), and ABSI is used to quantify the severity of visceral obesity. Similarly, a recent study revealed a relationship between VAI and Klotho protein levels in different cases, establishing the optimal VAI cut-off value (25). Likewise, the association between serum Klotho levels and ABSI in our study holds, which enriches the study of visceral obesity and Klotho protein.

Our study has some strengths. First, to the best of our knowledge, this is the first report of an association between visceral fat and serum anti-aging protein levels in humans. This provides new and practical insights into resistance or delay in aging, including appropriate weight control, especially visceral fat. Second, Klotho protein has a wide range of biological effects, indicating its important role not only in the mechanism of anti-aging but also in the occurrence and development of many diseases, which our study complements. Moreover, we used a multiethnic and large multiregional population based on the large population analysis from NHANES, which included a relatively large sample size of 11,070 Americans.

This study has some limitations. Due to the characteristics of the cross-sectional study design, the main limitation is the inability to establish a causal relationship between ABSI and Klotho concentration. Within a certain range, lower protein levels were associated with higher ABSI. Additionally, information on ABSI was obtained from the questionnaire. Some participants may have been reluctant to answer relevant questions for various reasons, resulting in missing data, while others may have omitted some information when answering the questionnaire, both of which inevitably lead to bias. Finally, although we attempted to account for confounding factors as much as possible in the adjusted models, there may still be potential confounding factors that have not been adjusted. Whether these factors affect the association between the two variables requires further research for confirmation.

### 4.1 Conclusion

Based on a nationally representative population, this study shows a negative correlation between ABSI and serum levels of the anti-aging protein Klotho in adults in the United States, with no nonlinear relationship.

## Data Availability

The datasets presented in this study can be found in online repositories. The names of the repository/repositories and accession number(s) can be found in the article/**Supplementary Material**.
